# Antiviral effects of Pediococcus acidilactici isolated from Tibetan mushroom and comparative genomic analysis

**DOI:** 10.3389/fmicb.2022.1069981

**Published:** 2023-01-10

**Authors:** Tianming Niu, Yuxin Jiang, Shuhui Fan, Guilian Yang, Chunwei Shi, Liping Ye, Chunfeng Wang

**Affiliations:** Animal Microecological Engineering Research Center, College of Animal Science and Technology, College of Veterinary Medicine, Jilin Agriculture University, Changchun, China

**Keywords:** rotavirus, probiotics, *Pediococcus acidilactici*, antiviral effect, whole genome

## Abstract

Rotavirus is one of the main pathogens that cause diarrhoea in young animals, and countless animals have died of rotavirus infection worldwide. Three strains of lactic acid bacteria isolated from Tibetan mushrooms were used to study the inhibition of rotavirus *in vitro* and *in vivo*. One part was to identify and study the biochemical and probiotic characteristics of three isolated lactic acid bacteria, and the other part was to evaluate the inhibitory effect on rotavirus *via in vivo* and *in vitro* experiments. The whole genome of the lactic acid bacteria with the best antiviral effect was sequenced, and the differences between them and the standard strains were analyzed by comparative genomic analysis, so as to provide a theoretical basis for exploring the antiviral effect of lactic acid bacteria.The three strains were identified as *Pediococcus acidilactici*, *Lactobacillus casei* and *Lactobacillus paracasei*. *Pediococcus acidilactici* showed good acid tolerance, bile salt tolerance, survival in artificial intestinal fluid, survival in gastric fluid and bacteriostasis. In *in vitro* experiments, pig intestinal epithelial cells cocultured with *Pediococcus acidilactici* exhibited reduced viral infection. In the *in vivo* experiment, the duodenum of mice fed *Pediococcus acidilactici* had extremely low numbers of virus particles. The total genome size was 2,026,809 bp, the total number of genes was 1988, and the total length of genes was 1,767,273 bp. The proportion of glycoside hydrolases and glycoside transferases in CAZy was 50.6 and 29.6%, respectively. The Metabolism function in KEEG had the highest number of Global and overview maps. Among the comparative genomes, *Pediococcus acidilactici* had the highest homology with GCF 000146325.1, and had a good collinearity with GCF 013127755.1, without numerous gene rearrangement events such as insertion, deletion, inversion and translocation. In conclusion, *Pediococcus acidilactici* was a good candidate strain for antiviral probiotics.

## Introduction

Rotavirus is one of the main pathogens that cause diarrhoea in infants and young animals. Approximately 215,000 infants and young children die from rotavirus infection worldwide each year, and most of these deaths occur in developing countries (Mm et al., 1997). At present, there is no specific drug for rotavirus, and vaccination with a live attenuated vaccine is the most effective way to prevent and control rotavirus-induced diarrhoea, but the vaccine acceptance rate in developing countries is relatively low, so the disease has not been controlled (Omatola et al., 2021; Song, 2021). Probiotics are a group of microorganisms that are beneficial to the host and can maintain the microecological balance of the host body, regulate the immune response, and reduce the occurrence of infectious diseases and intestinal inflammation. Therefore, probiotics are also an important means to prevent rotavirus infection. In recent years, an increasing number of studies have reported that probiotics can effectively shorten the duration of rotavirus-induced diarrhoea, reduce the incidence of diarrhoea and regulate intestinal homeostasis (Mao et al., 2016).

Tibetan mushroom, also known as Tibetan kefir, is the most common natural starter in Tibet, China. It is used to ferment milk at room temperature and has been eaten by Tibetans for hundreds of years. Tibetan mushrooms are rich in lactic acid bacteria, acetic acid bacteria, yeast and other probiotics (Gao and Zhang, 2019), which can produce high concentrations of lactic acid probiotic factors. As a probiotic resource, the Tibetan mushroom not only has rich nutrition but also has many beneficial functions, including the abilities to inhibit pathogenic bacteria (Sun et al., 2019), regulate body immunity, and exert anti-inflammatory and antioxidant effects (Tang et al., 2018). Therefore, Tibetan mushroom has high research value and significance. In clinical practice, Tibetan mushroom fermented milk has obviously alleviated diarrhea, so it is particularly important to explore the type of bacteria that can alleviate diarrhea and its antiviral effect.

An increasing number of probiotics have been reported in antiviral studies. Rather, I et al. used *Lactobacillus plantarum* as an adjuvant drug in the treatment of SARS-CoV-2 infection patients, inhibiting the replication of SARS-CoV-2, regulating the immune response, and reducing the levels of inflammatory factors such as IFN-ɑ, IFN-β, and IL-6. It has a good blocking effect on SARS-CoV-2 (Rather et al., 2021). Salaris,C et al. used *in vitro* experiments combining *Lactobacillus paracasei* with lactoferrin protein, which could significantly inhibit SARS-CoV-2 infection and enhance the ability of Caco-2 intestinal epithelial cells to resist SARS-CoV-2 (Salaris et al., 2021). Huang,S et al. found that metabolites of *Lactobacillus plantarum* (LPM) had a good inhibitory effect on porcine epidemic diarrhoea virus. After treating Vero cells with LPM metabolites, PEDV adsorption could be effectively prevented, the inflammatory response was reduced, and apoptosis of damaged cells was induced (Huang et al., 2021). Watanabe et al. showed that oral immunization of mice with *Enterococcus faecalis* could effectively reduce influenza virus infection, and specific antibodies against influenza virus were detected in the serum of the mice. Therefore, *Enterococcus faecalis* plays an important role in this process (Watanabe et al., 2021). Shin D et al. found that the *Lactobacillus plantarum* product developed in their study could effectively reduce the occurrence of rotavirus enteritis in children and inhibit the adhesion and proliferation of rotavirus in the small intestine (Shin et al., 2020).

In this study, we have isolated and identified three new lactic acid bacterial strains from Tibetan mushrooms and evaluated their probiotic properties, including acid resistance, bile salt resistance, survival in simulated artificial intestinal juice, survival in artificial gastric juice, and inhibition of the growth of pathogenic bacteria. The effects of the three strains on rotavirus replication and immune regulation have also tested *in vitro* and *in vivo* experiments. The best antiviral lactic acid bacteria have selected for all-cause sequencing, and their differences have analyzed by comparing with the model strains to explore their potential antiviral effects. Our goal has to develop the best antiviral lactic acid bacteria into a product for the prevention or treatment of diarrhea caused by rotavirus.

## Materials and methods

### Isolation and identification of lactic acid bacteria from traditional Tibetan mushrooms

Tibetan mushrooms (20 g) were added to 500 ml of sterilized milk and incubated at room temperature for 24 h. The milk could be fermented into a fermented milk product, which was subsequently fermented into a milky form. In this fermented milk, there were a large number of microbial strains from Tibetan mushrooms, and we used this fermented milk for lactic acid bacterium separation. After 100 μl of Tibetan mushroom fermented milk was serially diluted with 900 μl of phosphate-buffered saline (PBS), it was spread on De Man Rogosa Sharpe (MRS) agar and placed in an anaerobic culture incubator at 37°C for 18 h. After single colonies grew on the medium, a single colony was selected and placed in liquid MRS medium (Hurtado-Romero et al., 2021). After culture, only Gram-positive bacteria and catalase-negative bacteria were selected. Unless otherwise specified, the experiment was repeated three times.

Finally, three strains of Gram-positive bacteria with different colony morphologies were selected, and genomic DNA of the 3 g-positive bacterial strains was extracted. PCR amplification was carried out by using the 16S rDNA method. The general primers used for 16S rDNA amplification are shown in Table 1. The PCR system volume was 20 μl, including PCR enzyme mix (10 μl), the upstream primer and downstream primer (1 μl), the template (2 μl), and sterile water (6 μl). PCR conditions were as follows: 96°C for 3 min, followed by 30 cycles of 96°C for 1 min, 56°C for 30 s, and 72°C for 1 min, and finally 72°C for 7 min. After the PCR was completed, PCR products were subjected to agarose gel electrophoresis to observe the experimental results. The PCR products were then sent to Shanghai Shenggong Biological Co., Ltd. for sequencing, and the sequencing results were compared on NCBI GenBank. After comparison, the three strains were identified as lactic acid bacteria.

**Table 1:** Primer sequences and annealing temperatures for polymerase chain reactions.

Gene	Primer sequence (5`-3`)	Product size (bp)	Annealing temp. (°C)
IL-18	Forward: TGAAAACGATGAAGACCTGGAA	101	60
	Reverse: CCTGGTTAATGAAAAGGACTTGG		60
IL-6	Forward: ATGAGAAGTGTGAAAACAGCAAGG	294	60
	Reverse: CATTTGTGGTGGGGTTAGGG		60
IFN-β	Forward: TCGCTCTCCTGATGTGTTTCTC	82	60
	Reverse: AAATTGCTGCTCCTTTGTTGGT		60
TNF-α	Forward: GGCGTGAAGCTGAAAGACAAC	127	60
	Reverse: GGCTGATGGTGTGAGTGAGG		60
IL-1β	Forward: AAGTGGTGTTCTGCATGAGCTTT	125	60
	Reverse: CAGGGTGGGCGTGTTATCTT		60
IFN-α	Forward: ACCTCAGCCAGGACAGCAGTATC	121	60
	Reverse: TCGCAGCCCAGAGAGCAGATG		60
GAPDH	Forward: TGTGTCCGTCGTGGATCTGA	150	60
	Reverse: TTGCTGTTGAAGTCGCAGGAG		60
27F	Forward: AGAGTTTGATCMTGGCTCAG	1900	60
1492R	Reverse: GGTTACCTTGTTACGACTT		60

### Biochemical identification experiment

The three isolated lactic acid bacteria were inoculated into 10 solutions of biochemical identification reagents, including aescin, cellobiose, maltose, mannitol, salicin, sorbitol, sucrose, raffinose, inulin and lactose (biochemical reagents were purchased from Shandong Qingdao Haibo Biological Co., Ltd.). After anaerobic culture at 37°C for 18 h, the biochemical results were observed, and the data were statistically analyzed. At the same time, the isolated lactic acid bacteria were inoculated into a solution of nitrate reductant, and the experimental results were observed.

### Growth curve determination

The three isolated strains of lactic acid bacteria were added to MRS medium at a bacterial density of 1×10^7^ CFU/ml. Subsequently, a UV spectrophotometer was used to detect the OD value, which was measured at 0 h. Then, the corresponding OD values at 3 h, 6 h, 9 h, 12 h, 15 h, 18 h, 21 h, 24 h, and 36 h were detected, and the growth curve was drawn.

### Artificial gastric and intestinal fluids were simulated

The cultured strains were added to simulated artificial intestinal fluid and artificial gastric juice at a bacterial density of 1 × 10^7^ CFU/ml (artificial intestinal fluid and gastric juice were prepared in accordance with the Chinese pharmacopoeia). After 12 h of culture, MRS plate counting was performed after serial dilution, and the number of colonies was counted.

### Tolerance to different concentrations of bile salts and pH 2.5

Lactic acid bacteria (1 × 10^7^ CFU/mL) were added to the MRS liquid medium. After 18 h of anaerobic culture at 37°C, the bacteria were enriched, resuspended in PBS, and added to MRS medium at pH 2.5 for 12 h. The number of single colonies was counted by MRS plate counting. Similarly, resuspended lactic acid bacteria were added to MRS liquid medium with pig bile salt concentrations of 0.30, 0.50 and 1.00% for 12 h, and single colonies of treated lactic acid bacteria were counted.

### Inhibition of pathogenic bacteria

One of the most important tests of lactic acid bacteria is whether they can inhibit pathogenic bacteria. Only lactic acid bacteria that can inhibit pathogenic bacteria can be used in further applicantios. We selected *Escherichia coli*, *Salmonella typhimurium* and *Staphylococcus aureus* as pathogenic bacteria to test against the three strains of lactic acid bacteria. First, *Escherichia coli*, *Salmonella typhimurium*, and *Staphylococcus aureus* were plated on nutrient AGAR medium. Then, according to the drilling method, a 6 mm hole was introduced, and the three lactic acid bacteria were added to the holes. At the same time, the antibiotic group and the blank group were established, and the plates were placed in a 37°C anaerobic culture after 18 h. Finally, the size of the bacteriostatic ring was determined to be large or small. The diameter of the antibacterial ring was measured.

### Cells and viruses

We used pig intestinal epithelial cells for *in vitro* experiments. The cells were stored in the Animal Microecology Preparation Center of Jilin Agricultural University. In *in vitro* and animal experiments, we used porcine rotavirus (rotavirus is stored in the Animal Microecological Preparation Center of Jilin Agricultural University).

### *In vitro* antiviral experiment

We carried out *in vitro* anti-rotavirus experiments. First, we seeded 1 × 10^4^ IPEC-J2 cells in 6-well plates and then set up 6 experimental groups, namely, the PBS group, challenge group, antiviral drug group, *Pediococcus acidilactici* group, *Lactobacillus casei* group and *Lactobacillus paracasei* group. Next, 1×10^5^ CFU/ml lactic acid bacteria were added to IPEC-J2 cells and incubated for 2 h, and then gentamicin was added at a final concentration of 50 μg/ml. After incubation for 1 h, PBS was used for stringent washing 5 times. Then, the cells were infected with RV (virus titre of RV 100 TCID50); after infection for 12 h, then the total RNA of the supernatant and the cells was extracted and reverse transcribed into cDNA. Finally, fluorescence quantitative PCR was used to explore the inhibition of RV replication.

### Fluorescence quantitative PCR detection and analysis

We used absolute quantitative methods to conduct real-time PCR in a 20 μl reaction system using constructed plasmids as templates. The 20 μl system consisted of 10 μl of enzyme, 1 μl of upstream primer, 1 μl of downstream primer, 2 μl of template and 6 μl of sterile water. The reaction conditions were as follows: 52°C for 2 min, 95°C for 5 min, and 45 cycles of 95°C for 15 s and 58°C for 1 min. Then, the standard curve was drawn, and the experimental results were interpolated onto the standard curve to calculate the virus copy number for each treatment.

At the same time, a relative quantitative method was used to quantify the changes in the levels of cytokines produced by the cells under different treatments. The reaction system and reaction conditions were the same as those for the absolute quantitative method. Relative quantification of target genes was calculated using the 2^-ΔΔCt^ method (Zhao et al., 2020). The mRNA level of GAPDH was selected as the internal reference, the final results were calculated, and a histogram was drawn. The fluorescence quantitative primers are listed in Table 1.

### Antiviral experiment in mice

Specific pathogen-free (SPF) mice (Jeong et al., 2021) aged 3 weeks were used (the mice were from the Animal Center of Jilin Agricultural University), and 6 groups were set with 10 mice in each group, including the blank group, challenge group, antiviral drug group, *Pediococcus acidilactici* group, *Lactobacillus casei* group and *Lactobacillus paracasei* group. The immunization procedure was repeated three times after one insufflation of lactic acid bacteria and 2 days of rest. Finally, 100 TCID50 of rotavirus was administered continuously for 3 days. We measured the weight changes of mice 14 days after infection with the virus and plotted the weight change curve. Additionally, pathological sections of the small intestine and colon of mice in each group were made, and RNA of two intestinal segments was extracted. All the mice were finally euthanized, which was in line with ethical approval.

### Elisa

In the animal experiments, the serum and faeces of mice were collected before feeding bacteria, after feeding bacteria and 3 days after challenge. According to the instructions of the ELISA kits, IFN-β, IL-6, and TNF-α in serum and SIgA in faeces were quantified (ELISA kits were purchased from Jiangsu MEIMIAN Industry Co., Ltd.). For treatment of faeces, the faeces samples were weighed at low temperature, and then an equivalent amount of sterile PBS was added to dissolve the faeces. After overnight incubation at 4°C, the faeces samples were centrifuged at 12000 r/min for 2 min, and the supernatant was transferred. The concentration of tested samples was analyzed statistically, and a histogram was drawn.

### The intestinal tract of mice was examined by pathology

The duodenum and colon of mice under various treatments were collected, and the treated duodenum and colon were dyed with haematoxylin and eosin and then made into pathological sections for pathological examination. See the Supplementary materials for the specific steps.

### Whole genome sequencing

*Pediococcus acidilactici* was activated, and the bacteria were collected. The total bacterial DNA was extracted by using the bacterial DNA gene extraction kit and sent to Beijing Nuohe Source Technology Co., LTD for sequencing. After passing the library test, Pac Bio Sequel and Illumina Nova Seq PE150 sequencing were performed. After filtering and quality control of the original disembarkation data, SMRT Link V5.0.1 software was used to preliminarily assemble the original disembarkation data, and the assembled data were corrected to obtain the genome assembly data. The optimized assembly data were compared and analyzed with the assembly results, and the sequencing results were corrected. Glimmer V3.02 and Gene Mark S V4.3 software were used to predict the coding genes of the Mycococcus lactis genome. The Clusters of Orthologous Groups of proteins (COG) database was annotated by EGG NOG V4.5.1 software. KEGG was annotated through the Kyoto Encyclopedia of Genes and Genomes (KEGG) online database. The database of CAZy is annotated through rum-active En Zymes (CAZy) V6.0 software. Secondary metabolites were annotated by Anti SMASH V4.0.2 software.

### Comparative genome analysis

Phylogenetic trees of homologous single copy genes were constructed using IQ-Tree V1.6.12 software, and the TREE construction method was Maximum Likelihood. Mummer V4.0 analysis software was used for collinearity analysis with default software parameters. To calculate the similarity or evolutionary distance between different genomes for species classification and relative comparison. Therefore, by comparing the Average Nucleotide Identity (ANI), the genetic relationship of two genomes was compared at the Nucleotide level. In order to find out the conserved features of the genome, and thus the phenotypic variation of the genome, the relationship between the very conserved core genome and the number of genes in the pan genome was analyzed.

### Statistical analysis

Values are expressed as the mean ± standard deviation (SD). Statistical significance was assessed at *p* < 0.05 for all comparisons. Statistical analysis was performed with Statistical Product and Service Solutions (SPSS.19) statistical software.

## Results

### Results of isolation and identification of lactic acid bacteria

Single colonies on MRS solid medium were selected for Gram staining, and finally, three strains of Gram-positive Lactobacillus were identified. Based on observation, one strain was a Gram-positive coccus, and two were Gram-positive *Bacillus brevis*. According to 16S rDNA identification, the Gram-positive coccus was *Pediococcus acidilactici*, and the Gram-positive short bacilli were *lactobacillus casei* and *lactobacillus paracasei* (see the Supplementary material S1 for the comparison results, 200X).

### Biochemical identification experiment

The three isolated and identified lactobacillus strains (*Pediococcus acidilactici*, *Lactobacillus casei* and *Lactobacillus paracasei*) were inoculated with 10 biochemical identification reagents. The results of the experiment showed that *Pediococcus acidilactici* was positive for cellobiose, salicin, and inulin fermentation, while it was negative for aescin, maltose, mannitol, sorbitol, sucrose, raffinose and lactose fermentation. The biochemical identification results for *Lactobacillus casei* and *Lactobacillus paracasei* were the same. Both *Lactobacillus casei* and *Lactobacillus paracasei* could ferment aescin, cellobiose, maltose, mannitol, salicin, sorbitol, sucrose, inulin and lactose, while they were negative for raffinose fermentation. The results are shown in Table 2.

**Table 2:** Biochemical test.

Strain/biochemical reagent	Aescin	Cellobiose	Maltose	Mannitol	Salicin	Sorbitol	Sucrose	Raffinose	Inulin	Lactose	Reducing nitrate
*Pediococcus acidilactici*	−	+	−	−	+	−	−	−	+	−	+
*Lactobacillus casei*	+	+	+	+	+	+	+	−	+	+	−
*Lactobacillus paracasei*	+	+	+	+	+	+	+	−	+	+	−

(+) was positive, indicating a reaction. (−) was negative, indicating no reaction.

### Growth curve drawing

**Figure 1:**
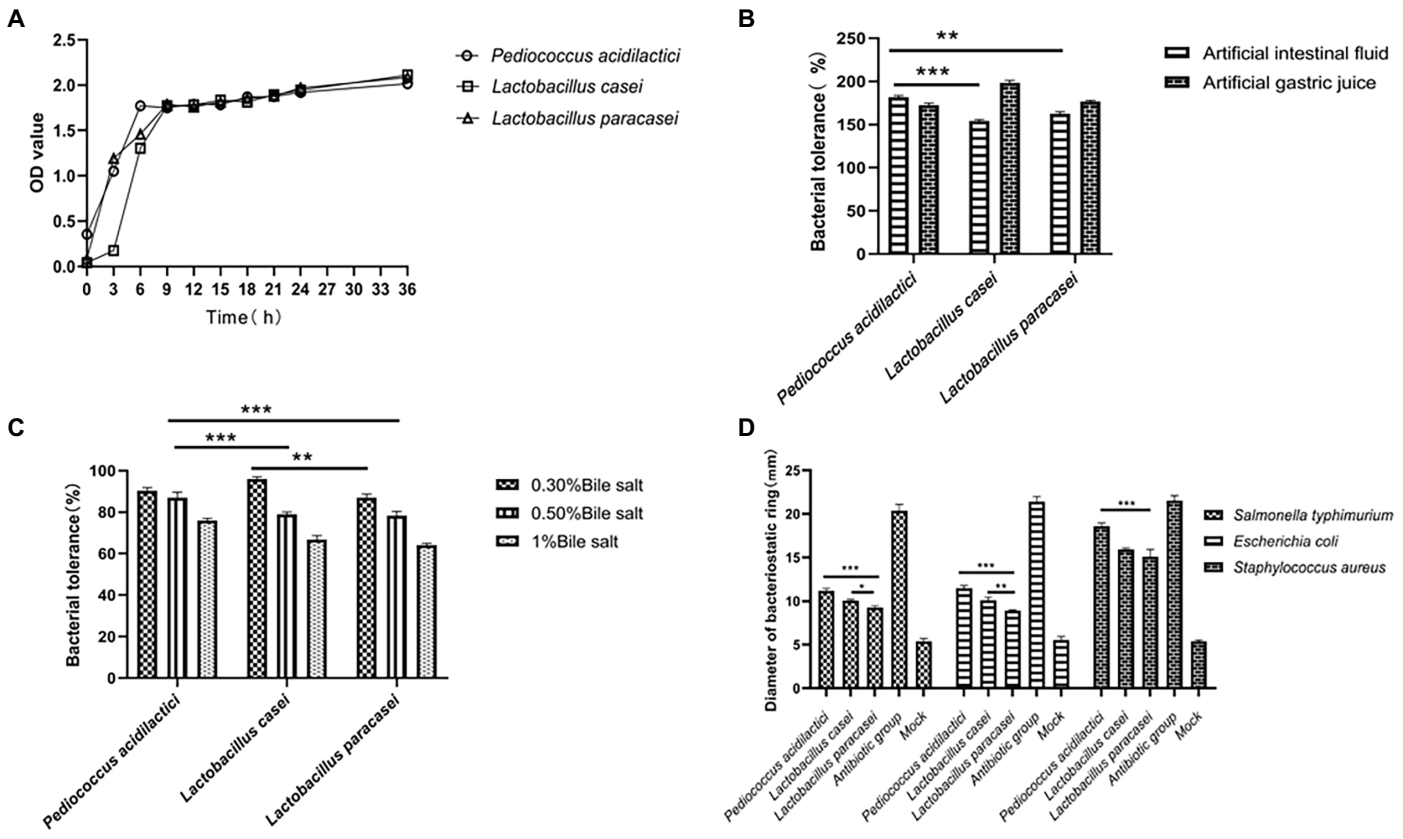
Growth characteristics and tolerance of isolated bacteria. **(A)** Growth curve of three isolated lactic acid bacteria. **(B)** Tolerance of three strains under artificial simulated intestinal fluid and gastric juice conditions. **(C)** Tolerance of three strains of bacteria under different concentrations of bile salt. **(D)** Inhibitory effects of three strains on three pathogens (the pathogenic bacteria were *Escherichia coli*, *Salmonella* and *Staphylococcus aureus*).

According to the growth of the three strains of lactic acid bacteria, a growth curve was drawn. The results showed that in the growth process of the three strains of lactic acid bacteria, the growth of the three strains was different in the logarithmic phase. The growth rate of *Pediococcus acidilactici* was higher than that of *Lactobacillus paracasei*, and that of *Lactobacillus casei* was the slowest. However, in the plateau phase, the growth of the three strains of lactic acid bacteria was relatively identical, as shown in Figure 1A.

### Artificial gastric and intestinal fluids were simulated

We compared the tolerance of the three strains of lactic acid bacteria by simulating the gastric juice and intestinal juice environments. In the gastric juice environment, the tolerance of *Pediococcus acidilactici* was significantly higher than that of *Lactobacillus casei* (*p* < 0.001) and that of *Lactobacillus paracasei* (*p* < 0.01). However, the tolerance of *Lactobacillus casei* in the intestinal fluid environment was significantly higher than that of *Pediococcus acidilactici* and *Lactobacillus paracasei* (*p* < 0.01). The results are shown in Figure 1B.

### Acid and bile salt tolerance results

We prepared MRS liquid medium with a pH of 2.5, added lactic acid bacteria at a concentration of 1 × 10^6^ CFU/ml to the medium to allow them to tolerate the conditions for 12 h, and then carried out counting and statistical analysis through the drop plate experiment. The results showed that the tolerance of *Lactobacillus paracasei* was stronger than that of *Pediococcus acidilactici* and *Lactobacillus casei* in the medium with pH = 2.5, and the tolerance percentage was 69%.

Pig bile salt was used to prepare MRS liquid media with bile salt concentrations of 0.30, 0.50 and 1%, and lactic acid bacteria at a concentration of 1 × 10^6^ CFU/ml were added to the MRS liquid media. After 12 h of culture, drop plate counting and statistical analysis were used. The results showed that the tolerances of *Pediococcus acidilactici* and *Lactobacillus casei* in MRS with 0.30% bile salt was similar, and both were stronger than that of *Lactobacillus paracasei*. The tolerance of *Pediococcus acidilactici* in MRS with 0.50% bile salt and 1% bile salt was higher than that of *Lactobacillus casei* and *Lactobacillus paracasei*. The experimental results are shown in Figure 1C.

### Inhibition of pathogenic bacteria

We selected *Escherichia coli*, *Salmonella typhimurium* and *Staphylococcus aureus* as the three pathogenic bacteria for the resistance testing of *Pediococcus acidilactici*, *Lactobacillus casei* and *Lactobacillus paracasei*. The antibacterial ability was determined by the diameter of the antibacterial ring. We measured the diameter of the bacteriostatic ring (mm), and the experimental results showed that the three strains of lactic acid bacteria showed good inhibitory effects on Salmonella, *Escherichia coli* and *Staphylococcus aureus*, among which *Pediococcus acidilactici* showed good inhibitory effects on the three pathogenic bacteria and was significantly more potent than *Lactobacillus casei* and *Lactobacillus paracasei* (*p* < 0.01). The experimental results are shown in Figure 1D.

### *In vitro* antiviral experiment

**Figure 2:**
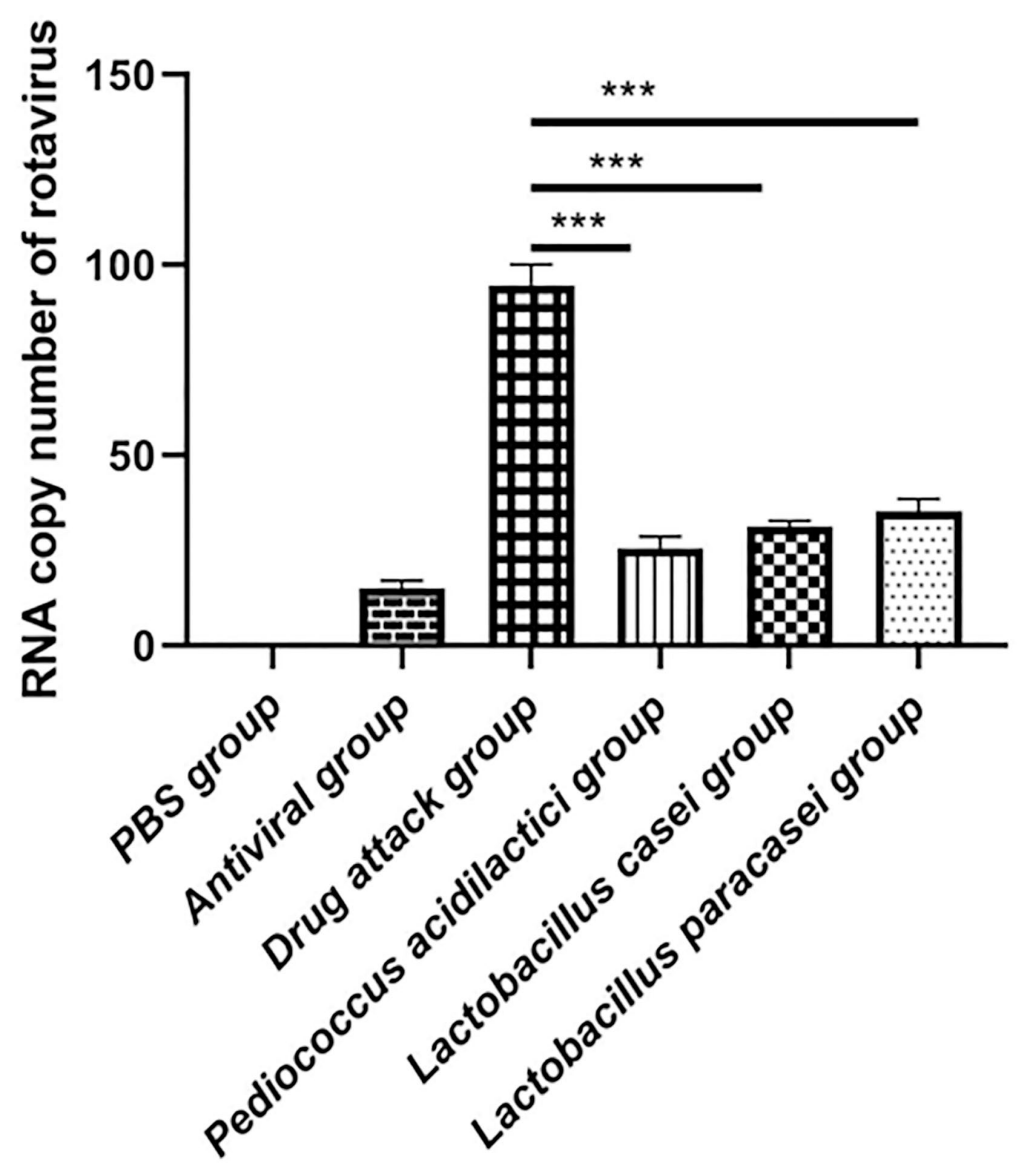
The number of virus copies was quantified in cultures of lactic acid bacteria and porcine intestinal epithelial cells.

In the *in vitro* assessment, we divided the experiment into 6 groups, They were PBS group, challenge group, antiviral drug group, *Pediococcus acidilactici* group, *Lactobacillus casei* group and *Lactobacillus paracei* group. The RNA of the virus was extracted and reverse transcribed into cDNA. The number of virions in cells treated by each condition was determined by absolute quantification. The experimental results showed that the virus copy number in the cells treated with the three strains of lactic acid bacteria was reduced. Compared with that in the challenge group, the virus copy number was significantly lower in the *Pediococcus acidilactici* group, *Lactobacillus casei* group and *Lactobacillus paracasei* group (*p* < 0.001), and the virus copy number under the *Pediococcus acidilactici* treatment was the lowest, indicating the best inhibitory effect, followed by that with *Lactobacillus casei*. The experimental results are shown in Figure 2.

**Figure 3:**
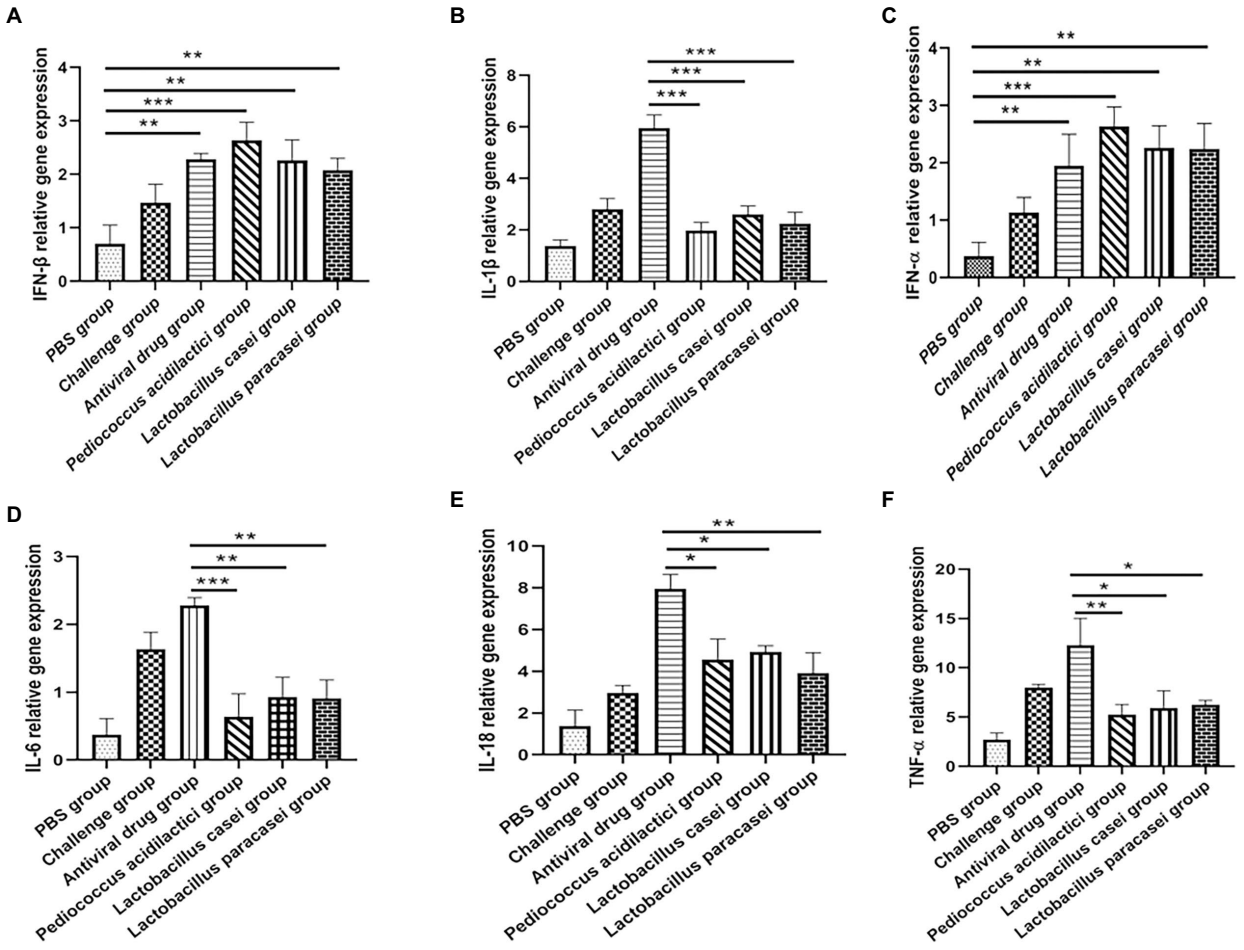
Lactobacillus and porcine intestinal epithelial cell cultures were used to quantify the cytokines produced by cells. **(A)**, IFN-β relative gene expression. **(B)**, IFN-α relative gene expression. **(C)**, IL-1β relative gene expression. **(D)**, IL-6 relative gene expression. **(E)**, IL-18 relative gene expression. **(F)**, TNF-α relative gene expression.

Total RNA was extracted from IPEC-J2 cells after each treatment, and the relative expression levels of IFN-ɑ, IFN-β, IL-1β, IL-6, IL-18, and TNF-ɑ were quantitatively determined. The experimental results showed that when quantifying IFN-ɑ and IFN-β in IPEC-J2 cell cultures, the relative expression levels of IFN-ɑ and IFN-β in the *Pediococcus acidilactici* group, *Lactobacillus casei* group, *Lactobacillus paracasei* group and the challenge group were significantly increased compared with those in the PBS group (*p* < 0.01), and the relative expression levels in the *Pediococcus acidilactici* group were the highest (Figures 3A,B). When quantifying IL-1β, IL-6, IL-18 and TNF-ɑ in IPEC-J2 cell culture, the relative expression levels of L-1β, IL-6, IL-18 and TNF-ɑ were significantly decreased in the *Pediococcus acidilactici* group, *Lactobacillus casei* group and *Lactobacillus paracasei* group compared with those in the challenge group (Figures 3C,F), among which the relative expression levels in the *Pediococcus acidilactici* group were the lowest. The experimental results are shown in Figure 3.

### *In vivo* antiviral experiment

**Figure 4:**
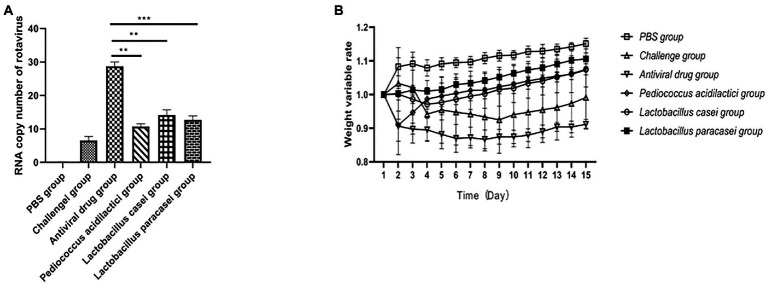
Lactic acid bacteria inhibit rotavirus replication *in vivo*. **(A)**, The number of viral RNA copies in the small intestine of mice after challenge. **(B)**, Weight loss rate of mice after challenge.

In *in vivo* experiments, we performed absolute quantitative comparisons of virus copy numbers in the duodenum of mice under different treatments. The results showed that immunization of mice with the three lactic acid bacteria strains could prevent rotavirus infection. The virus copy number in the duodenum of mice in the *Pediococcus acidilactici* group, *Lactobacillus casei* group and *Lactobacillus paracasei* group was significantly lower than that in the challenge group (*p* < 0.01). The virus copy number of mice treated with *Pediococcus acidilactici* was the lowest, indicating that *Pediococcus acidilactici* could effectively inhibit virus infection and replication. The experimental results are shown in Figure 4A.

After challenge, the body weight of the mice under different treatments changed significantly. The results showed that the body weight of the mice treated with the three strains of lactic acid bacteria gradually recovered after the second day after challenge, and the body weight of the mice immunized with *Lactobacillus paracasei* increased faster than that of the mice in the *Pediococcus acidilactici* group and *Lactobacillus casei* group, but there was no significant difference. The weight loss of mice in the challenge group was obvious, and the weight change of mice in the antiviral drugs group was also obvious. The experimental results are shown in Figure 4B.

### ELISA results

**Figure 5:**
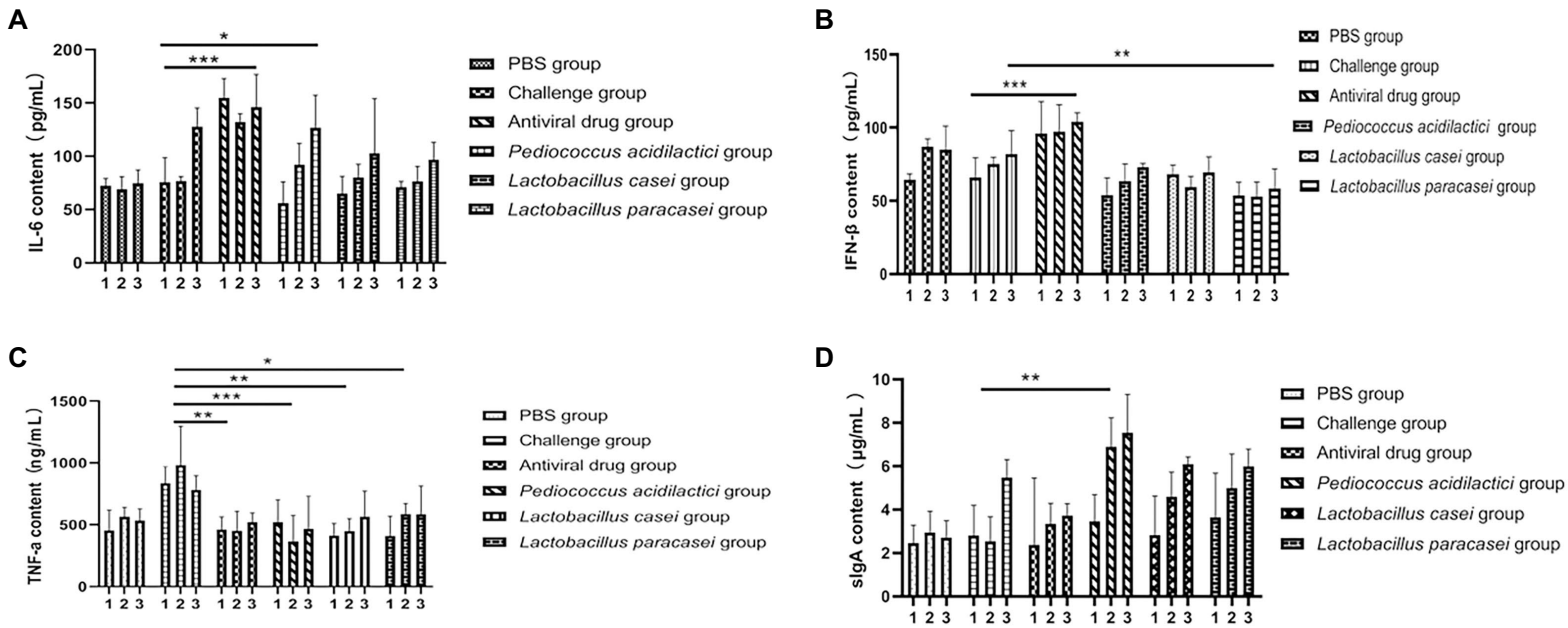
Cytokines and secretory sIgA in the blood of mice were detected by ELISA. **(A)** The change in IFN-β concentration in the blood of mice in three time periods. **(B)** The change of the concentration of IL-6 in the three stages. **(C)** The change in TNF-α concentration in the three stages. **(D)** Changes in the sIgA concentration in faeces (1 means before feeding lactic acid bacteria, 2 means after feeding Lactobacillus, 3 indicates after rotavirus infection).

We collected the blood and faeces of each group of mice before feeding with the bacteria, after feeding with the bacteria and after challenge and detected the levels of IFN-β, IL-6, TNF-α in serum and of SIgA in faeces by ELISA to study the changes. The results showed that the serum IFN-β content in the three lactic acid bacterium groups changed little after feeding with the bacteria and challenge but increased significantly in challenge group (Figure 5A). Compared with that in the blood of the PBS group, the IL-6 content in the blood of all treatment groups after challenge was significantly increased (*p* < 0.05; Figure 5B). When detecting TNF-ɑ in blood, compared with that in the challenge group, TNF-ɑ content in all treatment groups was significantly decreased (*p* < 0.01; Figure 5C). When detecting SIgA in faeces, the content of SIgA in faeces of the *Pediococcus acidilactici* group was significantly higher than that of the challenge group (*p* < 0.01; Figure 5D). The experimental results are shown in Figure 5.

### The pathological examination results

**Figure 6:**
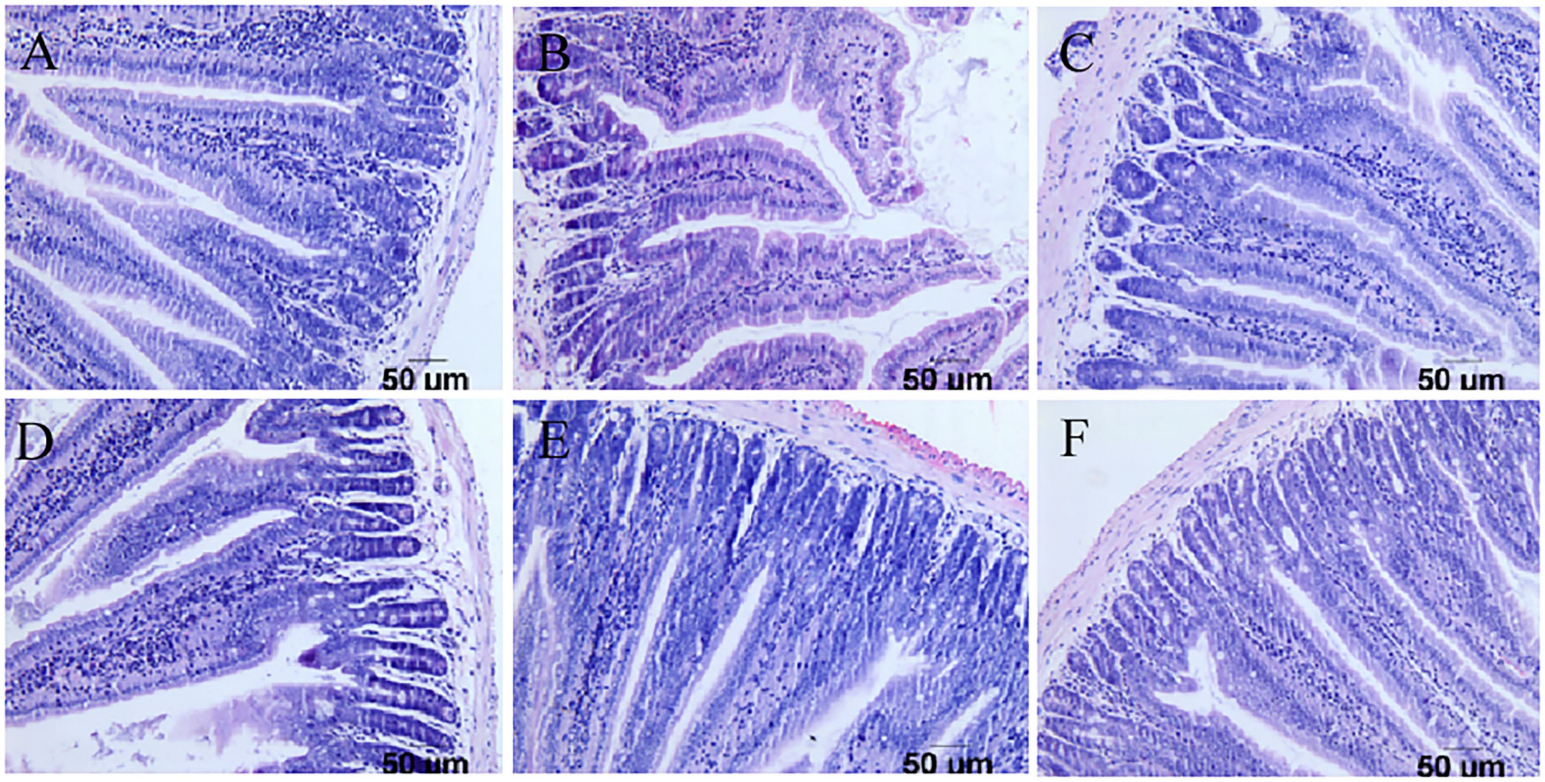
Pathological sections of the small intestine in mice. **(A)** Pathological sections of the small intestine in the PBS group. **(B)** Pathological sections of the small intestine in the challenge group. **(C)** Pathological sections of the small intestine in the antiviral drug group. **(D)** Pathological sections of the small intestine in the *Pediococcus acidilactici* group. **(E)** Pathological sections of the small intestine in the *Lactobacillus casei* group. **(F)** Pathological sections of the small intestine in the *Lactobacillus paracasei* group.

**Figure 7:**
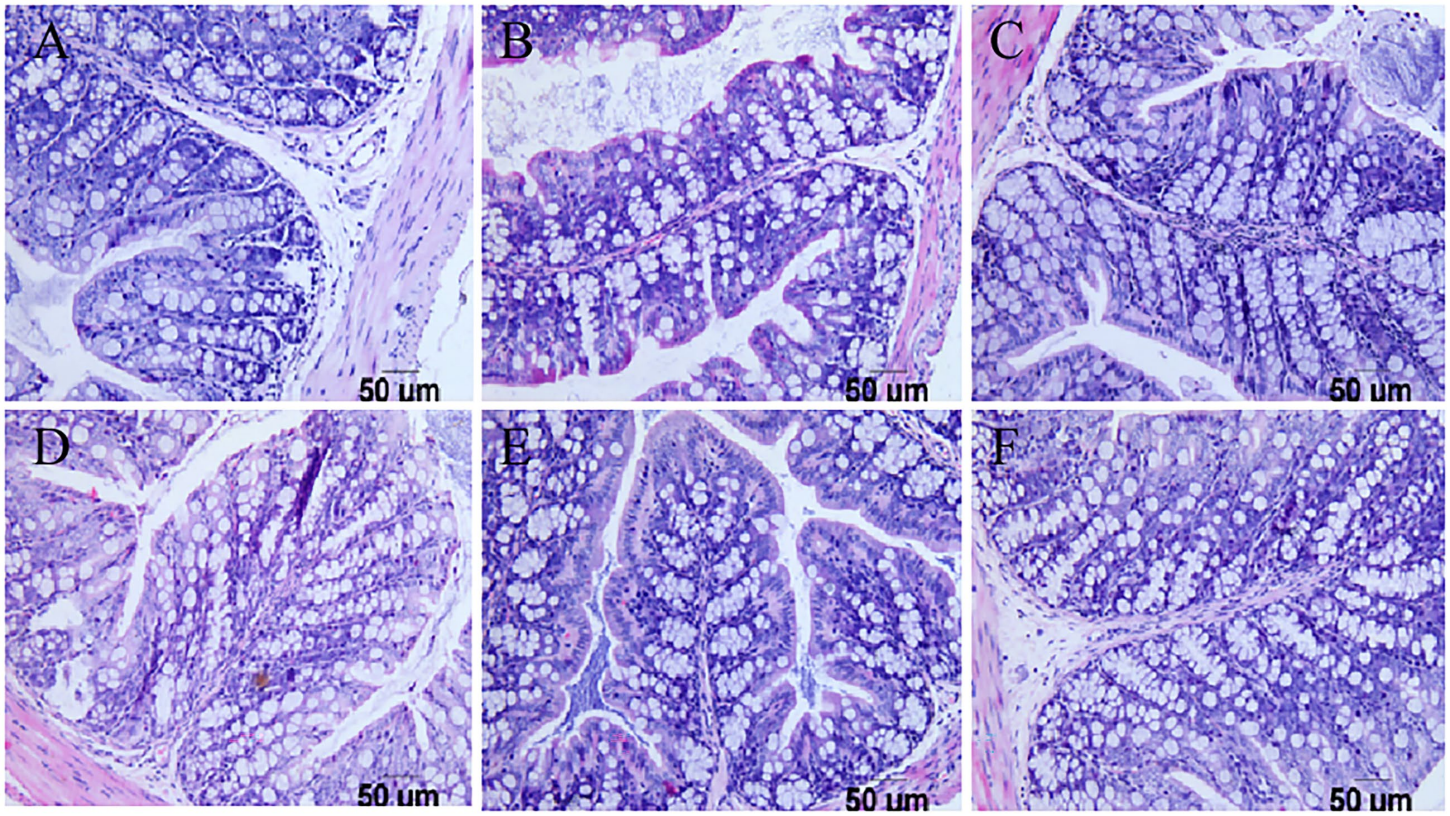
Pathological sections of the large intestine in mice. **(A)** Pathological sections of the large intestine in the PBS group. **(B)** Pathological sections of the large intestine in the challenge group. **(C)** Pathological sections of the large intestine in the antiviral drug group. **(D)** Pathological sections of the large intestine in the *Pediococcus acidilactici* group. **(E)** Pathological sections of the large intestine in the *Lactobacillus casei* group. **(F)** Pathological sections of the large intestine in the *Lactobacillus paracasei* group.

Pathological examination of the duodenum and colon in mice showed that in the challenge group, the virus destroyed the integrity of the duodenal villi of the mice and appeared at the top of the intestinal villus injury. Inflammatory cell infiltration was accompanied by interstitial oedema, inflammatory cell infiltration in the colon submucosa was visible, and goblet cell numbers increased at the same time. However, there were no obvious pathological changes in the three groups with the lactic acid bacteria, and the intestinal villi of mice in the *Pediococcus acidilactici* group were relatively intact. The experimental results are shown in Figures 6, 7.

### Whole genome sequencing results

**Figure 8:**
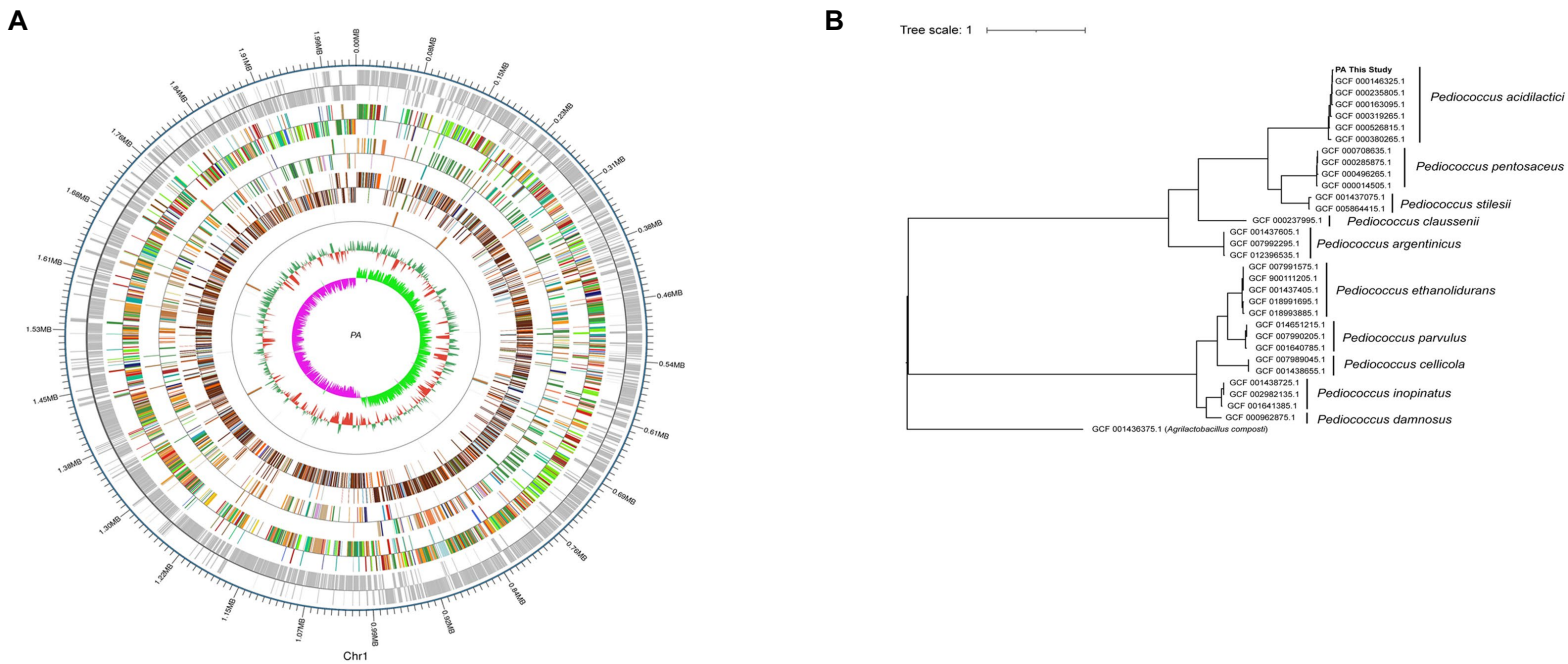
Whole gene sequencing of *Pediococcus acidilactici.* **(A)** A complete diagram of bacteria. **(B)** Comparison of evolutionary trees of *Pediococcus acidilactici.*

The whole genome of *Pediococcus acidilactici* was composed of a ring structure with chromosome 2,026,809.The number of genes was 1988, the total length of genes was 1,767,273 bp, and the average length of genes was 889 bp. Using CRISPRdigger for genome CRISPR prediction, the number of CRISPR was 6, with a full length of 1,070 bp and an average length of 178.333 bp. The number of tRNA was 57, with an average length of 75 bp and a full length of 4,310 bp (Figure 8A). By comparison and analysis of evolutionary trees, PA of *Pediococcus acidilactici* was similar to GCF 000146325.1 (Figure 8B).

**Figure 9:**
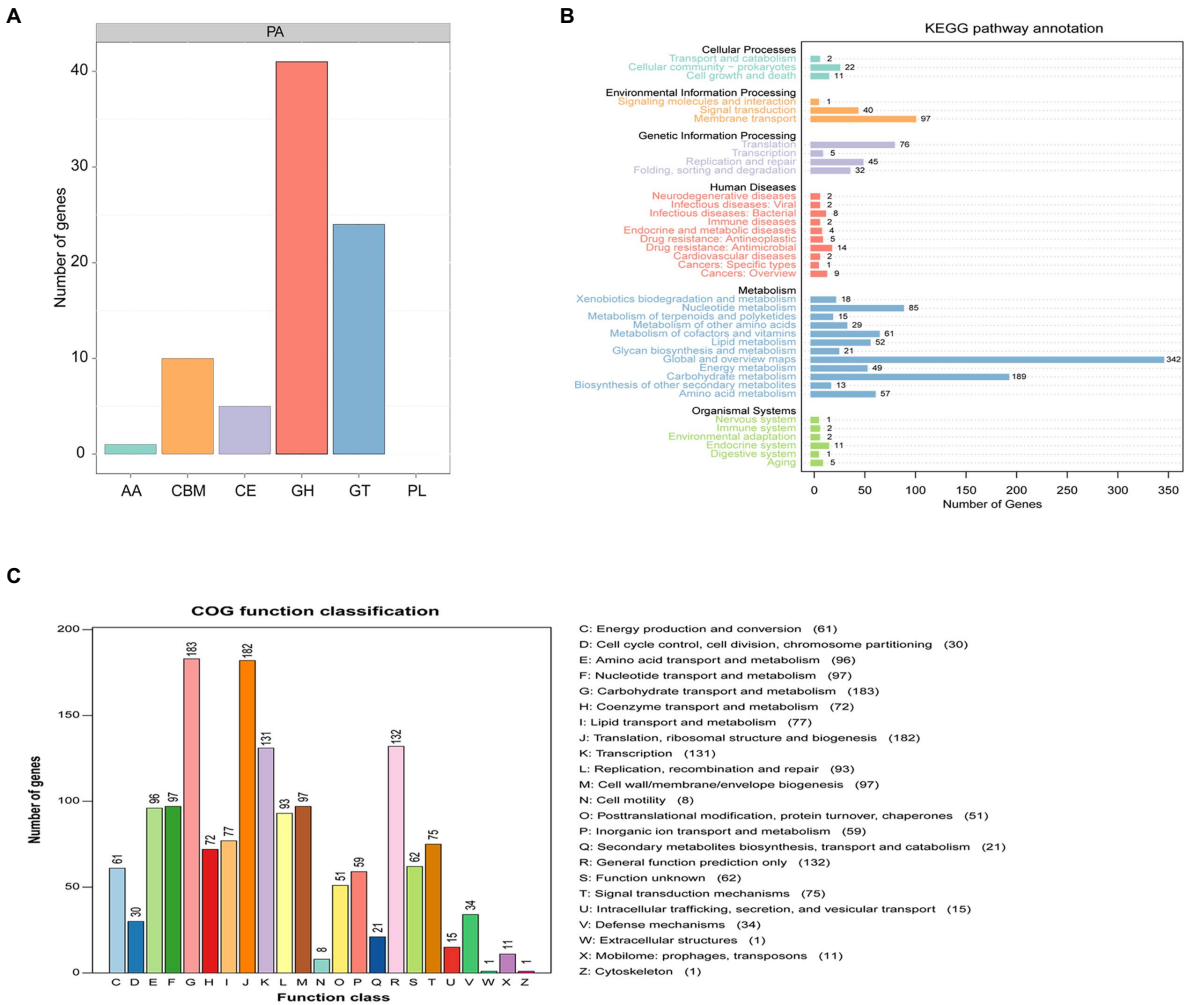
Functional prediction of *Pediococcus acidilactici.* **(A)** CAZy analysis of *Pediococcus acidilactici.* **(B)** Analysis of KEGG signaling pathway in *Pediococcus acidilactici.* **(C)** COG function prediction analysis of *Pediococcus acidilactici.*

A total of 81 genes were annotated to 5 active enzymes by CAZy of PA of *Pediococcus acidilactici*, among which 50.62% of genes were annotated as glycoside hydrolases (GH), 29.63% were annotated as glycosyl transferases (GT). 6.17% genes were annotated as carbohydrate esterases(CE), 4.83% genes were annotated as auxiliary activity(AA). And 12.35% genes were annotated as rum-binding modules (CBM; Figure 9A). A total of 1,333 genes were annotated to functional information in KEGG annotation of *Pediococcus acidilactici*. The number of genes related to cellular process was 2.63%, the number of genes related to metabolism was 69.84% and the number of genes related to human diseases was 3.68%. The number of genes related to genetic information processing was 11.85%, and the number of genes related to biological systems was 1.65%. The number of genes associated with environmental information processing was 10.35%. In the KEGG functional annotation, the most genes were related to metabolic pathways, among which the proportion of Global and Overview maps was 25.66%, followed by the proportion of metabolism 14.18% (Figure 9B); COG annotation of *Pediococcus acidilactici* has a total of 1910 genes annotated to four COG functions, Its functions were mainly concentrated in Carbohydrate transport and metabolism (9.58%), Ribosomal structure and Transcription(6.86%) and biogenesis(6.91%). In addition, 1.10% of the genes were annotated to be related to secondary metabolites biosynthesis (transport and catabolism) (Figure 9C).

### Comparative genomic results

**Figure 10:**
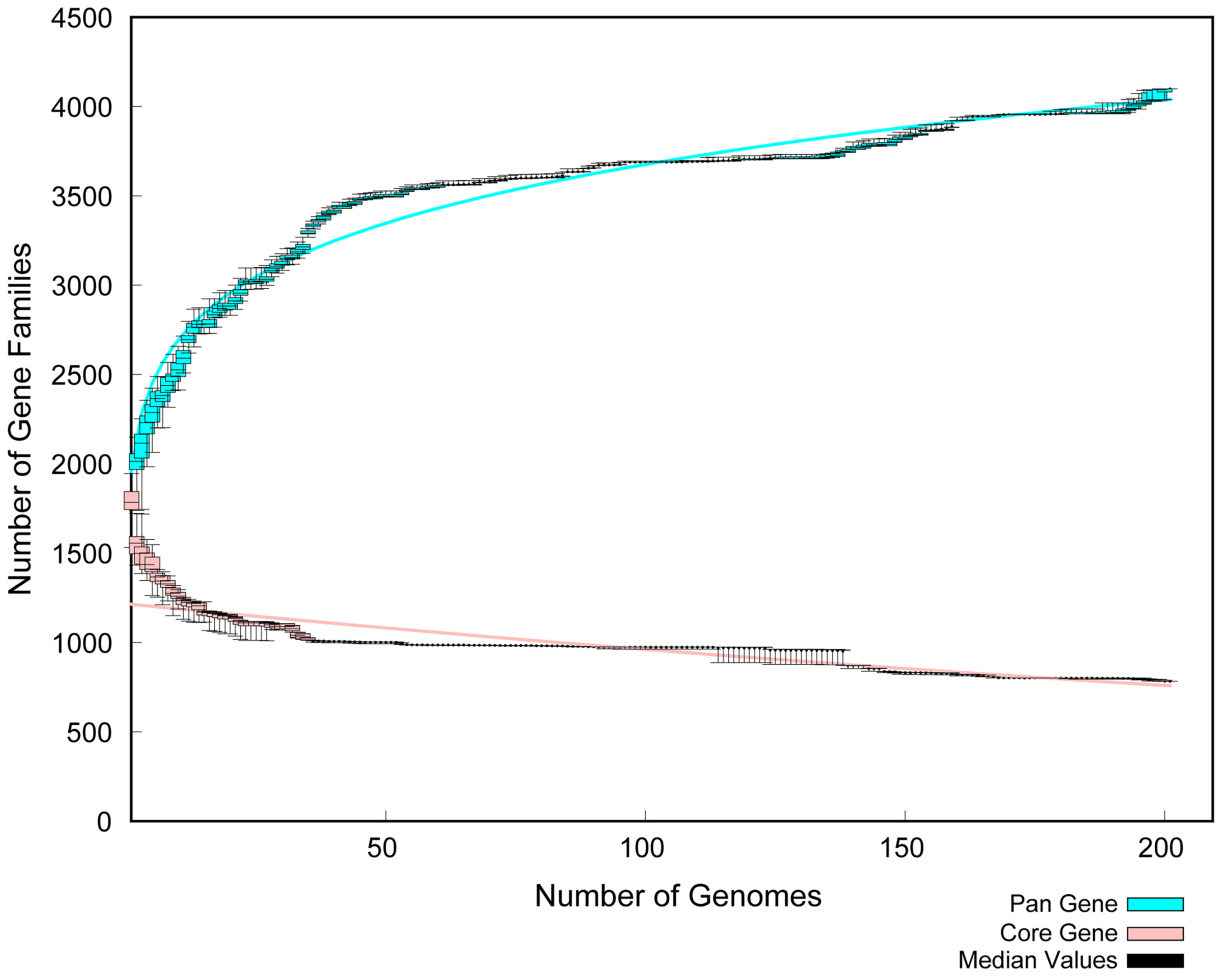
Comparative analysis of Pan Gene and Core Gene in *Pediococcus acidilactici.*

**Figure 11:**
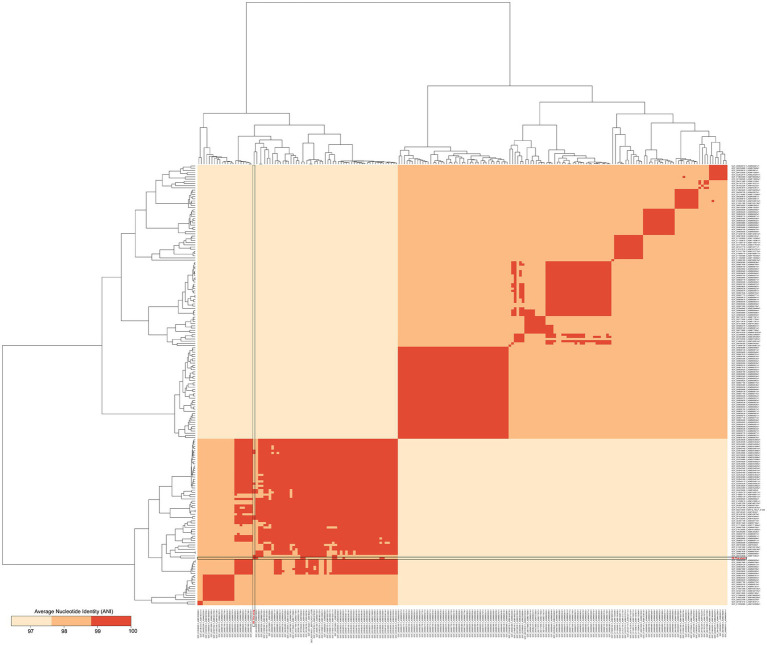
Nucleotide levels were compared between two genomic relatedness.

**Figure 12:**
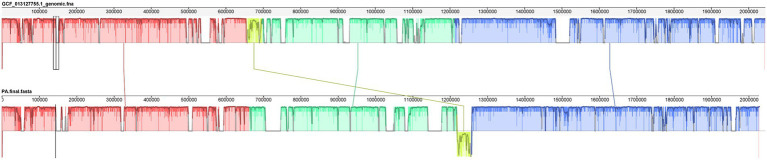
Collinearity analysis of *Pediococcus acidilactici* and GCF-013127755.1.

The whole genome of *Pediococcus acidilactici* was compared. In the pan-genome analysis, with the increase of the number of genes, the total number of genes in the pan-genome gradually increased, and the increase amplitude gradually decreased, while the number of core genome gradually decreased, and tended to be stable (Figure 10). In order to assess the relatability of the species, the average nucleotide identity analysis (ANI) showed that the ANI values of the 100 isolates were all above 97%, which was greater than the threshold of 96% for conspecific identification. It indicated that these strains belonged to *Pediococcus acidilactici*, and there was no subspecies (Figure 11). In order to explain the evolutionary relationship and multiploidy events among strains, the collinearity analysis was used to compare *Pediococcus acidilactici* with the GCF-013127755.1-genomic. Fna strain with high homology. The experimental results showed that gene insertion occurred in the red base sequence, while the yellow base sequence caused and translocated. However, for most base sequence comparisons, there was a good collinearity relationship (Figure 12).

## Discussion

Tibetan mushrooms have existed in China for many years and are called kefir in the West. Studies have reported that Tibetan mushrooms are extremely rich in lactic acid bacteria. Different types of fermented milk and different geographical locations have different structures of lactic acid bacteria, with some yeast as the dominant microorganism and some lactobacillus as the dominant bacterium. Therefore, researchers research results on Tibetan mushrooms are also inconsistent (Georgalaki et al., 2021). Some researchers isolated *Lactobacillus paracasei* from Tibetan kefir and found that the extracellular polysaccharide produced by it had good probiotic properties (Bengoa et al., 2021). It has been reported that *Saccharomyces cerevisiae* isolated from Tibetan mushrooms can prevent and treat gastrointestinal and other infectious diseases and can be used as an excellent fermentation preparation for production (Ansari et al., 2021). In this study, three strains of lactic acid bacteria, *Pediococcus acidilactici*, *Lactobacillus casei* and *Lactobacillus paracasei*, were isolated from Tibetan mushrooms, and their physiological and biochemical characteristics, probiotic properties and antiviral properties were studied. The inhibitory effects of the three strains on rotavirus were compared *in vitro* and *in vivo*.

Adikari, AMMU et al. isolated several strains of lactic acid bacteria from traditional fermented milk gel and evaluated their morphological, biochemical, physiological and probiotic properties (Adikari et al., 2021). In this study, three strains of lactic acid bacteria isolated from Tibetan mushrooms were sequenced by 16S rDNA sequencing, and the results confirmed *Pediococcus acidilactici*, *Lactobacillus casei* and *Lactobacillus paracasei* after comparison. There are no literature reports that *Pediococcus acidilactici* has been isolated from Tibetan mushrooms. Therefore, three strains of lactic acid bacteria were tested for their resistance to acid, bile salt, artificial gastric juice and artificial intestinal juice. In the acid resistance test, *Lactobacillus paracasei* showed a strong tolerance; under the condition of pH 2.5, it still had 69% tolerance. In the article of Falfan, Cotes RN et al., *Lactobacillus paracasei* also showed excellent acid resistance (Falfán-Cortes et al., 2021) In the bile salt tolerance test, *Pediococcus acidilactici* showed good tolerance, which was stronger than that of *Lactobacillus casei* and *Lactobacillus paracasei*. In the experiment assessing tolerance to artificial gastric juice, *Pediococcus acidilactici* could tolerate artificial gastric juice, and the loss of *Pediococcus acidilactici* in gastric juice was low. *Lactobacillus casei* showed strong tolerance in the artificial intestinal fluid experiment. *Pediococcus acidilactici* has a good inhibitory effect on *Escherichia coli*, *Salmonella typhimurium*, and *Staphylococcus aureus*, and its inhibitory ability was stronger than that of *Lactobacillus casei* and *Lactobacillus paracasei. Pediococcus acidilactici* isolated by Pei et al. had a significant inhibitory effect on *Salmonella typhimurium* (Pei et al., 2021).

In the *in vitro* experiment, under different treatments of IPEC-J2 cells, the copy number of virions in each group was compared to determine the inhibitory effect on virion proliferation. Compared with other treatments, *Pediococcus acidilactici* showed a strong inhibitory effect. It has been reported that *Lactobacillus casei* can prevent rotavirus adhesion on the MA104 cell surface (Olaya Galán et al., 2016; Fernandez-Duarte et al., 2018), and *Lactobacillus paracasei* can also inhibit virus replication. This is the first time that *Pediococcus acidilactici* has been shown to inhibit the virus, which may be related to the physiological characteristics of *Pediococcus acidilactici*. In the *in vivo* experiment, the rotavirus particle copy number in the duodenum of mice in the *Pediococcus acidilactici* group was the lowest, indicating that immunization with *Pediococcus acidilactici* could effectively prevent virus infection. In the statistical analysis of the body weight loss rate, lactic acid bacteria could effectively alleviate the weight loss caused by virus infection in mice and gradually promote body weight gain (Glubokova et al., 2021). Through the duodenum and colon pathological examination for each group of mice, we found that rotavirus can destroy the integrity of the mouse duodenum villi, appearing at the top of the intestinal villus damage; cause a small amount of inflammatory cell infiltration accompanied by tissue interstitial oedema and inflammatory cell infiltration in the colon submucosa visible; and increase the number of goblet cells (Liu et al., 2013; Tada et al., 2016). However, there were no obvious pathological changes induced by the three *lactobacillus* strains, and the intestinal villi of mice in the *Pediococcus acidilactici* group were relatively intact (Wu et al., 2013). Overall, the lactic acid bacteria isolated from Tibetan mushrooms had an inhibitory effect on the virus, among which *Pediococcus acidilactici* had the best inhibitory effect. In animal experiments, the levels of cytokines in blood and SIgA in the faeces of mice in each group were detected. After immunization with lactic acid bacteria, the changes in IFN-β levels in blood were not significant, but the levels of IL-6 increased, those of TNF-ɑ decreased, and those of SIgA in faeces showed an upwards trend, indicating that lactic acid bacteria can activate T cells. IL-6 can activate the lymphokines produced by T cells and fibroblasts, make the precursor of B cells become antibody producing cells, and cooperate with colony stimulating factors to promote the growth and differentiation of original bone marrow derived cells, and enhance the cleavage function of natural killer cells. In this experiment, the level of IL-6 increased significantly, which may be a factor for the role of *Pediococcus acidilactici* in the antiviral process. These effects of reducing inflammatory factor levels and increasing nonspecific antibody content to address viral infection were similar to those reported in the literature (Xie et al., 2021).

With the reduction of sequencing cost and the rapid development of sequencing technology, whole genome sequencing has become the most concise, effective, and rapid powerful tool to discover specific genes and explore the mechanism of action. Singh et al. compared the genome and phenotype of *Acinetobacter baumannii* AB030 with the highly resistant pathogen LAC-4 and found that AB030 contained many genes related to antibiotic resistance and virulence that were absent from LAC-4 (Singh et al., 2020). In this study, the second - and third-generation sequencing technologies were used to perform fine sequencing, analysis and functional annotation of the whole genome of *Pediococcus acidilactici* strain, and to analyze the biological characteristics and gene function of the strain at the molecular level.The collinearity analysis of the genome showed that the differences between the isolated *Pediococcus acidilactici* and GCF-013127755.1 strains were small, but there were still a small number of translocations and inversions, indicating that under long-term selection pressure, the strains expanded genes in the local collinear regions through gene horizontal transfer or directed evolution to add new functions and better adapt to the complex and variable environment.

*Pediococcus acidilactici*, *Lactobacillus casei*, and *Lactobacillus paracasei* isolated from Tibetan mushrooms have good probiotic properties and inhibitory effects on rotavirus infection. This study provided a feasible scheme for further understanding the infection process of rotavirus and the mechanism by which probiotics such as *Pediococcus acidilactici* inhibit virus replication.

## Data availability statement

All data generated or analyzed in this study are included in the article (and its supplementary information file) published here in. 16S rDNA sequencing data have been uploaded to NCBI, and GenBank submitted: OM390598.1, OM390600.1, OM390599.1. Pig rotavirus was donated by Professor Ren Xiaofeng of Northeast Agricultural University. The number is DN30209. *Escherichia coli* (ATCC 25922), *Salmonella typhimurium* (ATCC 14028) and *Staphylococcus aureus* (ATCC 25923).

## Ethics statement

The experiment was conducted in strict accordance with animal welfare and ethics guidelines.

## Author contributions

Dr. Niu TM designed this research and completed most of the experimental work. Master Jiang YX wrote the first draft of the manuscript, Master Fan SH, assisted in completing most of the experimental work. Teachers Yang GL, Shi CW, Ye LP, Wang CF provided theoretical guidance and technical support for this experiment. All authors have read and approved the manuscript.

## Funding

This work was supported by the National Natural Science Foundation of China (31941018, 32072888, and U21A20261), China Agriculture Research System of MOF and MARA (CARS35), Science and Technology Development Program of Jilin Province (YDZJ202102CXJD029, 20190301042NY, and 20220202057NC) and the Science and Technology Project of the Education Department of Jilin Province (JJKH20220366KJ).

## Conflict of interest

The authors declare that the research was conducted in the absence of any commercial or financial relationships that could be construed as a potential conflict of interest.

## Publisher’s note

All claims expressed in this article are solely those of the authors and do not necessarily represent those of their affiliated organizations, or those of the publisher, the editors and the reviewers. Any product that may be evaluated in this article, or claim that may be made by its manufacturer, is not guaranteed or endorsed by the publisher.
