# Supplementary material for: Antiviral effects of Pediococcus acidilactici isolated from Tibetan mushroom and comparative genomic analysis
Source: Front Microbiol. 2023 Jan 10;13:1069981. doi: 10.3389/fmicb.2022.1069981 (PMC9871908; doi:10.3389/fmicb.2022.1069981)
Supplement: Supplementary file 1 [file Data_Sheet_1.docx]

**[Antiviral effects of Pediococcus acidilactici isolated from Tibetan mushroom and comparative genomic analysis](https://review.frontiersin.org/Document/DownloadPDF?articleId=1069981&siteId=314&userId=1713368&roleId=17)**

Tianming Niu, Yuxin Jiang, Shuhui Fan, Guilian Yang, ChunWei Shi*, Liping Ye*, Chunfeng Wang*

College of Veterinary Medicine, College of Animal Science and Technology, Jilin Provincial Engineering Research Center of Animal Probiotics, Jilin Provincial Key Laboratory of Animal Microecology and Healthy Breeding, Key Laboratory of Animal Production and Product Quality Safety of the Ministry of Education, Jilin Agricultural University, Changchun, China

Chun-Feng Wang, College of Veterinary Medicine, College of Animal Science and Technology, Jilin Provincial Engineering Research Center of Animal Probiotics, Jilin Agricultural University, 2888 Xincheng Street, Changchun 130118, China. E-mail: wangchunfeng@jlau.edu.cn, Tel./fax: +86 43184533426.

Liping Ye, College of Veterinary Medicine, College of Animal Science and Technology, Jilin Provincial Engineering Research Center of Animal Probiotics, Jilin Agricultural University, 2888 Xincheng Street, Changchun 130118, China. E-mail: yeliping1114@163.com.

ChunWei Shi, College of Veterinary Medicine, College of Animal Science and Technology, Jilin Provincial Engineering Research Center of Animal Probiotics, Jilin Agricultural University, 2888 Xincheng Street, Changchun 130118, China. E-mail: shichunwei@jlau.edu.cn.


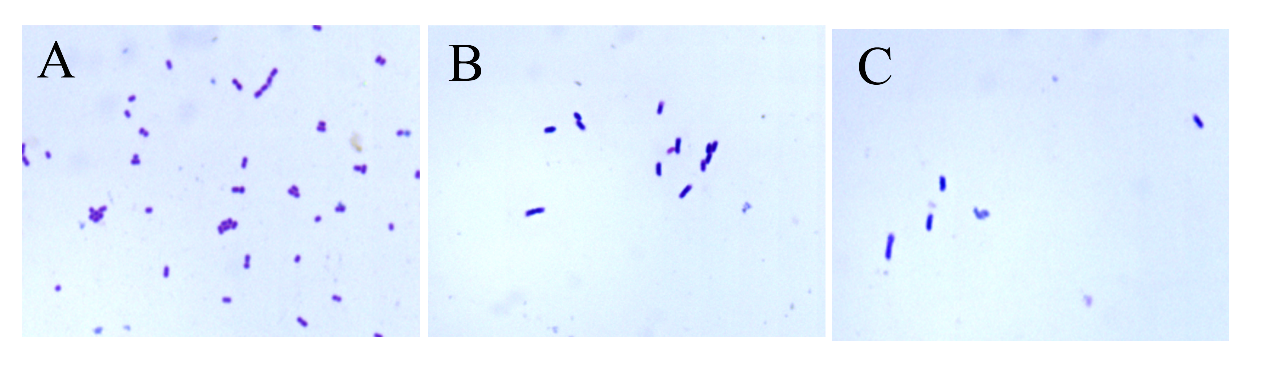


Supplementary Figure 1：Gram staining diagram(200X).

A：Pediococcus acidilactici.B：Lactobacillus casei.C：Lctobacillus paracasei.
